# LAMC2 regulates proliferation, migration, and invasion mediated by the Pl3K/AKT/mTOR pathway in oral

**DOI:** 10.32604/or.2023.029064

**Published:** 2023-06-27

**Authors:** FAYU SHAN, LANLAN LIANG, CHONG FENG, HONGBAO XU, ZIROU WANG, WEILI LIU, LINGLING PU, ZHAOLI CHEN, GANG CHEN, XINXING WANG

**Affiliations:** 1 Department of Environmental Medicine, Tianjin Institute of Environmental and Operational Medicine, Tianjin, 300070, China; 2 School and Hospital of Stomatology, Tianjin Medical University, Tianjin, 300050, China

**Keywords:** LAMC2, OSCC, Autophagy, PI3K/AKT/mTOR pathway, 3-Methyladenine, Rapamycin

## Abstract

**Background:**

Oral squamous cell carcinoma (OSCC) is a common malignant tumor. Recently, Laminin Gamma 2 (LAMC2) has been shown to be abnormally expressed in OSCC; however, how LAMC2 signaling contributes to the occurrence and development of OSCC and the role of autophagy in OSCC has not been fully explored. This study aimed to analyze the role and mechanism of LAMC2 signaling in OSCC and the involvement of autophagy in OSCC.

**Methods:**

To explore the mechanism by which LAMC2 is highly expressed in OSCC, we used small interfering RNA (siRNA) to knock down LAMC2 to further observe the changes in the signaling pathway. Furthermore, we used cell proliferation assays, Transwell invasion assays, and wound-healing assays to observe the changes in OSCC proliferation, invasion, and metastasis. RFP-LC3 was used to detect the level of autophagy intensity. A cell line-derived xenograft (CDX) model was used to detect the effect of LAMC2 on tumor growth *in vivo*.

**Results:**

This study found that the level of autophagy was correlated with the biological behavior of OSCC. The downregulation of LAMC2 activated autophagy and inhibited OSCC proliferation, invasion, and metastasis via inhibiting the PI3K/AKT/mTOR pathway. Moreover, autophagy has a dual effect on OSCC, and the synergistic downregulation of LAMC2 and autophagy can inhibit OSCC metastasis, invasion, and proliferation via the PI3K/AKT/mTOR pathway.

**Conclusions:**

LAMC2 interacts with autophagy to regulate OSCC metastasis, invasion, and proliferation via the PI3K/AKT/mTOR pathway. LAMC2 down-regulation can synergistically modulate autophagy to inhibit OSCC migration, invasion, and proliferation.

## Introduction

Oral squamous cell carcinoma (OSCC) is a common head and neck squamous cell carcinoma (HNSCs) in the world [1] and has a strong invasion and metastasis capability, which contributes to the low overall five-year survival rate of patients with OSCC [2]. According to the NCBI, there are an estimated 54,000 new cases of HNSC, and as many as 11,230 deaths attributed to this disease in 2022 (http://seer.cancer.gov). Typically, OSCC is treated with surgery, radiotherapy, and chemotherapy. Despite integrated sequential therapy strategies and chemical agents (Cispla), patients with OSCC continue to be associated with a high mortality rate in most tumor populations. Therefore, patients with OSCC must be receive highly effective treatment strategies [3,4].

Laminin is the major non-collagenous component of the basement membrane and is a type of extracellular matrix glycoprotein. Moreover, laminins are involved in various biological processes, including cell adhesion, metastasis, signal transduction, and metastasis [5]. Laminin gamma 2 chain (LAMC2) is one laminin chain [6] that is abnormally expressed in pancreatic [7], gastric [8], oral [9], lung [10], cervical [11], esophageal [12], and thyroid cancer [13]. Increasing evidence indicates that the high invasive potential and distant metastasis of tumor cells is dependent on LAMC2 expression, which is closely associated with tumor metastasis, recurrence, and patient mortality [14–16]. Since studies investigating the involvement of LAMC2 in OSCC are still in the initial stages, the specific mechanism by which LAMC2 affects the biological behavior of OSCC has not been fully elucidated [17]. Therefore, LAMC2 should be further examined to determine how it affects OSCC.

The PI3K/AKT/mTOR pathway signaling pathway plays an important role in tumors [18]. The PI3K/AKT/mTOR signaling pathway is abnormally expressed in some common tumors, thereby affecting the EMT process in tumors or promoting tumor metastasis, invasion, and proliferation [19]. In addition, the PI3K/AKT/mTOR signaling pathway is closely related to autophagy. Previous reports indicate that autophagy is regulated by the PI3K/AKT/mTOR signaling pathway to regulate tumor migration, invasion, and proliferation [20].

One cellular phenomenon that consumes internal or extracellular substances to promote cell survival is the process of autophagy [21]. Although autophagy exists widely in tumor cells, its complex role in tumors has not been fully explored. Autophagy has both pro-tumor and anti-tumor effects. When autophagy fluctuates during homeostasis (protective autophagy), the tumor is not adversely affected and its growth is promoted. However, when the level of autophagy exceeds a cell’s tolerance, autophagy (cytotoxic/nonprotective autophagy) can inhibit tumor survival [22]. Moreover, the level of autophagy has been shown to exhibit differential effects on tumors, even in different stages of the same tumor.

During the early stage of tumor development, autophagy can inhibit the activation of protooncogenes and tumor growth. However, autophagy can provide tumor cells with energy by converting damaged intracellular organelles and excessive proteins into nutrients [23–25]. Collectively, these findings indicate that the role of autophagy in tumor is highly complex. Therefore, the role of autophagy in OSCC should be further explored.

To date, no studies have reported any association between LAMC2 and autophagy. We hypothesized that LAMC2 interacts with autophagy and influences the ability of OSCC to metastasize, invade, and proliferate. To this end, we investigated how LAMC2 and autophagy affect OSCC metastasis and invasion. In this study, we found that LAMC2 and autophagy exhibit some subtle relationships.

## Materials and Methods

### Bioinformatics analysis

The tumor data from The Cancer Genome Atlas (TCGA) was analyzed using the GEPIA database (gepia.cancer-pku.cn) and linkedomics database (www.linkedomics.org). Briefly, the expression levels of specific genes between different tumors were analyzed using the Gene Expression Profile and Expression on Box Plots in the databases. A KEGG signal pathway enrichment analysis of specific genes was carried out by linkedomics database.

### Cells culture

Human normal oral epithelial cells (HNOECs), SCC-15, sCC-25, and CAL-27 cells were cultured in complete medium (DMED [Gibco]) containing 10% fetal bovine serum (FBS, Invitrogen, California, USA), penicillin, and streptomycin (Solarbio, Beijing, China) at 37°C in 5% CO_2_, 95% air. HNOECs were purchased from Guide Chem (https://china.guidechem.com, Zhejiang, China) and SCC-15, SCC-25, and CAL-27 cells were obtained from ATCC (www.atcc.org, USA).

### siRNA transfection

Negative controls and small interfering RNA (siRNA) oligos targeting LAMC2 were purchased from Gene Pharma (Shanghai, China). Transfection of cal-27 cells with siRNA was performed in accordance with manufacturer’s protocol. Cal-27 cells at 70% confluency was transfected with siRNA-mate (Gene Pharma, Shanghai, China) and 20 nM siRNA. After 72 h, WB or RT-qPCR analysis were used to observe the level of protein and gene expression. The oligo sequence of siRNA is shown in Table 1.

**Table 1:** si-RNA sequences

	Sense (5′–3′)	Antisense (5′–3′)
Si-NC	UUCUCCGAACGUGUCACGUTT	ACGUGACACGUUCGGAGAATT
Si-LAMC2-1919	GCAGGUGUUUGAAGUGUAUTT	AUACACUUCAAACACCUGCTT
Si-LAMC2 -2593	GCCACAAGAUUAGCAGAAATT	UUUCUGCUAAUCUUGUGGCTT
Si-LAMC2 -3504	GCUGGAGUUUGACACGAAUTT	AUUCGUGUCAAACUCCAGCTT

### Lentiviral infection

Lentivirus-encapsulated plasmids targeting microtubule-associated protein 1 light chain 3 (LC3) were used to detect the intensity of autophagy. Lentivirus-encapsulated interfering plasmids targeting LAMC2 were provided by Gene Pharma (Shanghai, China) and used to screen for LAMC2 in cal-27 cell lines that can be sustainably silenced. The siRNA oligo sequences are shown in Table 1. Briefly, 1 × 10^4^ cells were seeded into a six-well plate. After a 24-h incubation, the virus stock solution was mixed with medium and added to the six-well plate. After 12 h, replace the medium was replaced with fresh medium. After an additional 24 h of incubation under normal conditions, the transfection group was screened with puromycin (1 μg/μL) for 3 days. Next, 0.5 μg/μL puromycin was used for further screening for 2 days. A fluorescence microscope was used to observe the intensity of autophagy. The obtained cells exhibiting stably LAMC2-downregulated expression were used for subsequent *in vivo* experiments.

### Monodansylcadaverine (MDC) staining assay

Monodansylcadaverine assay kit (MDC; Solarbio, Beijing, China) was used to detect the formation of intracellular autophagosomes. A density of 1 × 104 untreated and si-NC or si-LAMC2 transfected cells were seeded into six-well plate. After 1 day, the cells were stained with 50 μM MDC for 15 min. A fluorescence microscope was used to obtain images of the samples.

### Western blot analysis

The cells were washed before adding phenylmethanesulfonyl fluoride (PMSF; Solarbio, Beijing, China) in RIPA Lysis Buffer (Solarbio, Beijing, China) for 30 min. The mixture was centrifuged for 10 min and the concentration was determined after removing the precipitate. The tumor tissue was washed twice and homogenized on ice. The mixture was centrifuged for 10 min and the concentration was determined after removing the precipitate. Cells (20 µg/well) or tissue lysates (40 µg/well) were added to each well and separated by electrophoresis on 4% to 20% SDS-polyacrylamide gels. The proteins were transferred to polyvinylidene fluoride (PVDF; Millipore, USA) membranes using electrophoresis and incubated with the following primary antibodies: LAMC2 (1:1000, Cat. no. ab274376, Abcam, Shanghai, China), PI3K (1:1000, Cat. no. ab151549, Abcam, Shanghai, China), E-cadherin (1:1000, Cat. no. 3195s, Cell Signaling Technologies, Shanghai, China), Vimentin (1:1000, Cat. no. 9856, Cell Signaling Technologies, Shanghai, China), Snail (1:1000, Cat. no. ab216347, Abcam, Shanghai, China), AKT (pan) (1:2000, Cat. no. 4691S, Cell Signaling Technologies, Shanghai, China), p-AKT (1:2000, Cat. no. 4060S, Cell Signaling Technologies, Shanghai, China), P70S6K (1:1000, Cat. no. 9202S, Cell Signaling Technologies, Shanghai, China), p-P70S6K (1:1000, Cat. no. 9204S, Cell Signaling Technologies, Shanghai, China), mTOR (1:1000, Cat. no. 2983s, Cell Signaling Technologies, Shanghai, China), p-mTOR (1:1000, Cat. no. 5536s, Cell Signaling Technologies, Shanghai, China), p62 (1:2000, Cat. no. ab109012, Abcam, Shanghai, China), Beclin-1 (1:1000, Cat. no. ab207612, Abcam, Shanghai, China), LC3 (1:1000, Cat. no. 14600-1-AP, Proteintech, Wuhan, China) or GAPDH (1:10000, Cat. no. AP0063, Bioworld, Nanjing, China). After 12 h, the membranes were incubated with a secondary antibody (1:10000, Cat. no. BS13278, Bioworld, Nanjing, China). The membranes were visualized using AI680 Images (Cytiva, USA).

### Immunohistochemistry (IHC) analysis

According to standard protocols for histological analysis, IHC analysis was performed as follows: tissue sections were soaked in phosphate balanced solution (PBS; Solarbio, Beijing, China) with Tween (Solarbio, Beijing, China) and subsequently blocked with serum (Boster Biological Technology, Wuhan, China.). The serum on the sections was removed and the primary antibody was added. The negative control tissues were added with PBS overnight at 4°C. The following day, the samples were removed from the refrigerator and rewarmed for 15 min. The samples were rinsed with PBS and rinsed with water. The sections were soaked and rinsed in PBS with Tween three times. After adding the secondary antibody, the samples were incubated for 30 min and washed with PBS. A hematoxylin dye solution was added for 2 min, rinsed with distilled water, a color separation solution was added, and then rinsed three times with water. The slides were sequentially dehydrated in 100% absolute ethanol. A microscope was used for observation and photography. The following primary antibodies were used: LAMC2 (1:300, Cat. no. ER65562, HuaAn Biotechnology, Hangzhou, China), E-cadherin (1:200, Cat. no. 3195s, Cell Signaling Technologies, Shanghai, China), LC3 (1:600, Cat. no. 14600-1-AP, Proteintech, Wuhan, China). Secondary antibody IgG H&L (1:1000, Cat. no. ab150077, Abcam, Shanghai, China).

### Real-time quantitative PCR assay

A total RNA extraction kit Sangon Biotech (Shanghai, China) was used in accordance with the manufacturer’s instructions. The cDNA was obtained using Veriti (Thermo Scientific, China). cDNA and A28134 (QuantStudio® 5 Real-Time PCR Instrument, Singapore) according to the manufacturer’s instructions. The relative quantity of the obtained results was evaluated using the comparative 2^−ΔΔCT^ method. The primers are listed in Table 2.

**Table 2:** Primer sequences

	Sense (5′–3′)	Antisense (5′–3′)
LAMC2	TGGATGCAGTA CAGATGGTGATT	GAGCTGGAAGG TTGTGGGTT
GAPDH	AATGGGCAGC CGTTAGGAAA	GCCCAATACGAC CAAATCAGAG

### Clone formation assay

Following transfection, untreated or transfected SCC-15 cells (1 × 10^4^ cells/mL) were seeded into 60-mm dishes and cultured in a cell incubator for two weeks. 4% formaldehyde was used to fix the cells for 15 min and 1 × Giemsa (Solarbio, Shanghai, China) was used to stain cells for 30 min. The size of the cloned cells was quantified by Image J (version 1.8.0, National Institutes of Health) software.

### Cellular proliferation assay

Cellular proliferation was detected using Cell Counting Kit-8 (DOJINDO, Japan). A total of 1 × 10^3^ cells were seeded into 96-well plates. At 24, 48, and 72 h, after removing the original medium and adding serum-free medium, 10 μL working solution was added to the wells to be detected and incubated for 1.5 h. The results were then obtained at 450 nm by Molecular Devices (SpectraMax® M5, USA).

### Transwell invasion assay

A Corning 24-well Transwell plate (8.0 Micron, Corning, USA) was used to detect cell invasion. The cell suspension (200 μL, 1 × 10^4^ cell/mL) was seeded into the upper chamber and placed in 24-well plates containing 20% FBS. After 2 days, the cells remaining on the upper filter were scraped off and the chamber was washed twice with PBS. After fixing the cells with 4% formaldehyde for 15 min, 1 × Giemsa (Solarbio, Shanghai, China) was used to stain the cells for 30 min at room temperature.

### Wound healing assay

This wound healing ability of CAL-27 cells was detected by a scratch test. When the cells had reached close to 100% confluency, 1 mL of the cell tips were used to create gaps between the cells, and after removal of the suspended cells, serum-free medium was added and culture was continued in the incubator for 72 h before photographing.

### Analysis of tumor growth in nude mice

To determine the effect of LAMC2 on OSCC growth *in vivo*, We selected 5-week female athymic mice for the experimental analysis. We randomly divided the mice into Control, si-NC, and si-LAMC2 groups (8 mice in each group). The cells used in animal experiments were comprised of the stable strains screened in Section 2.4 that could stably silence LAMC2. A total of 5 × 10^6^ cells were subcutaneously injected into the skin of the mice. Tumors were measured every 3 days until they were visible to the naked eye. Finally, the tumors were collected after the mice were euthanized (volume = D/2 × d^2^, (D = length, d = width)).

Animal experimental procedures were approved by the Tianjin Institute of Environmental and Operational Medicine Animal Ethics Committee and animals were cared in full accordance with the protocol.

### Statistical analysis

Appropriate statistical treatment of the data is essential. GraphPad Prism 9 was used to analyze the experimental data. The data were processed after obtaining the data of three experiments. Error bars represent the SEM. A two-tailed Student’s *t*-test or one-way ANOVA was used to analyze differences between groups. A one-way ANOVA was used to analyze multiple groups. A Tukey’s multiple comparisons test was performed to obtain the relevant *p* values. When *p* < 0.05, the analysis of experimental data was considered to be statistically significant.

## Results

### LAMC2 is abnormally expressed in HNSC and highly expressed in OSCC cell lines

**Figure 1:**
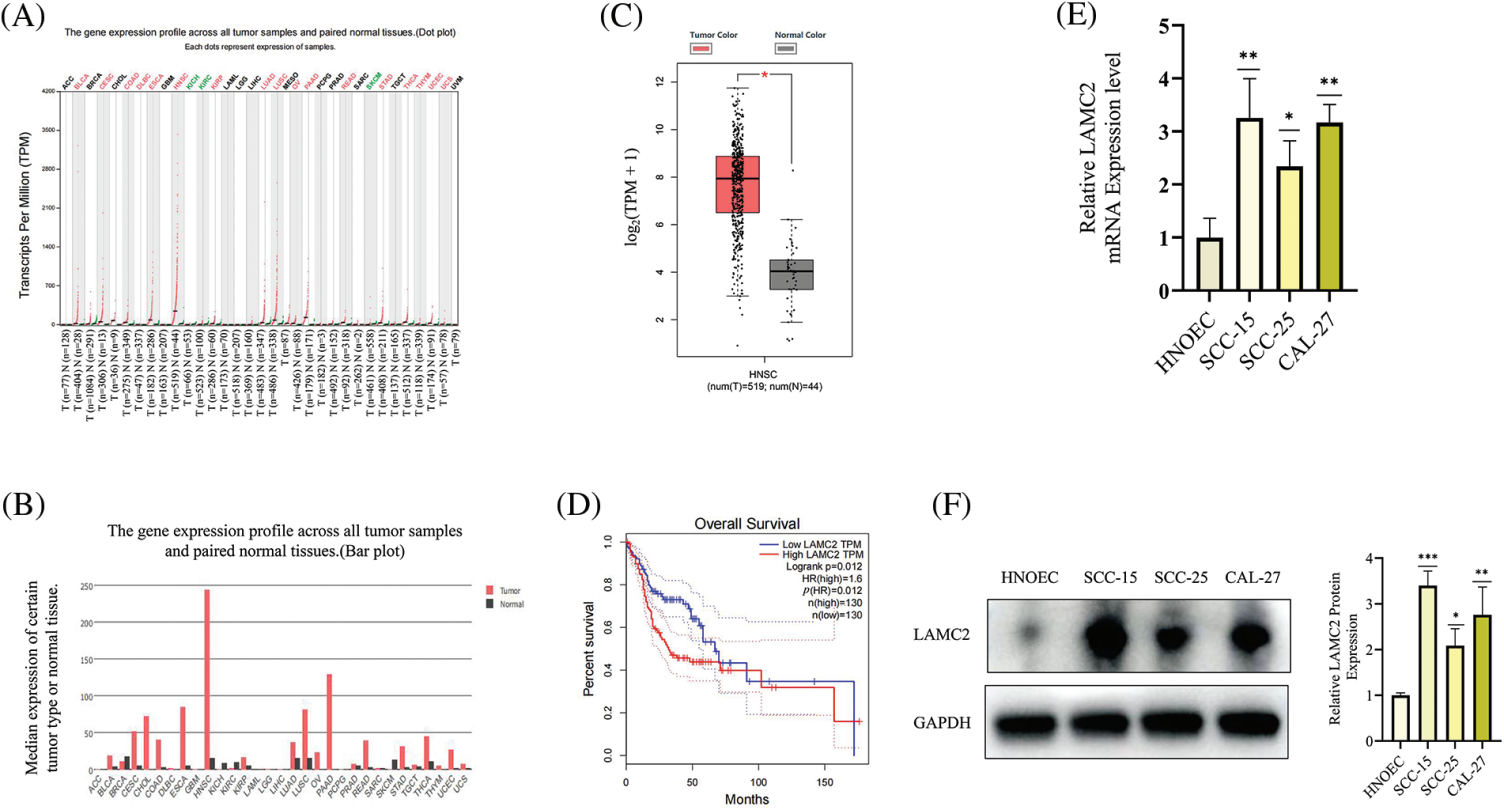
LAMC2 is highly expressed in HNSC and OSCC cell lines. (A–C) The GEPIA database shows the level of LAMC2 expression in different tumors. The levels of LAMC2 expression were abnormally elevated in HNSC patients compared to that of normal tissues (tumor tissues, n = 519; normal tissues, n = 44). (D) Patients with high and low LAMC2 expression display different survival curves. The survival rate was significantly reduced in patients with high LAMC2 expression compared to patients with low LAMC2 expression tumors. (E, F) LAMC2 expression in HNOEC and different OSCC cell lines. Each experiment was conducted at least three times and at least three sets of data were collected from each experiment for statistical analysis (*p < 0.05; **p < 0.01; ***p < 0.001).

The HNSC data were analyzed using the GEPIA (gepia.cancer-pku.cn) database. Using a single gene analysis, LAMC2 was highly expressed in HNSC compared with other cancer types (Figs. 1A and 1B). Moreover, LAMC2 was highly expressed in tumor tissues compared with normal tissues, exhibiting a significant difference (Fig. 1C). There was also a significant difference in the survival analysis between patients with high and low LAMC2 expression. The survival rate of patients with high LAMC2 expression was significantly lower than that of patients with low LAMC2 expression (Fig. 1D). In addition, we used a Western blot (WB) and RT-qPCR to analyze the expression of LAMC2 in HNOEC and three OSCC cell lines. Compared with HNOEC, LAMC2 was highly expressed at both the gene and protein level in three OSCC cell lines (Figs. 1E and 1F). Based on these results, the Cal-27 cell line exhibiting a moderate level of LAMC2 expression was selected for subsequent experiments.

### OSCC tumor proliferation, metastasis, invasion, and epithelial-mesenchymal transition (EMT) are inhibited by LAMC2 downregulation

**Figure 2:**
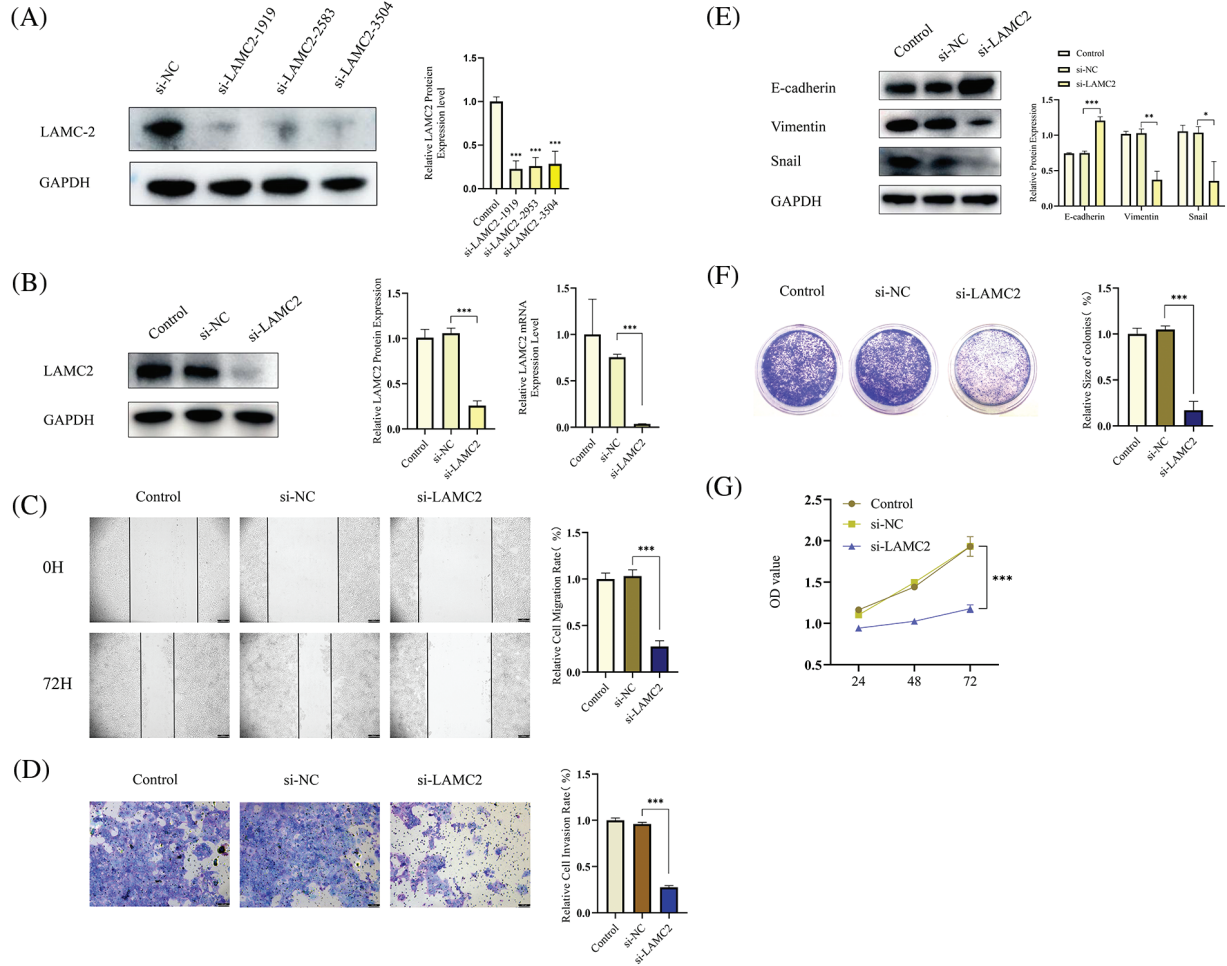
LAMC2 downregulation suppresses cell proliferation, metastasis, and invasion of OSCC tumors and inhibits the occurrence of EMT. (A, B) WB and RT-qPCR shows the level of LAMC2 protein and mRNA expression, respectively, following a LAMC2 knockdown using siRNA in cal-27 cells. (C, D) Wound healing assays (Original magnification, ×50) and Transwell invasion (Original magnification: ×100) were used to observe the ability of cal-27 cells metastasis and invasion ability after an LAMC2 knockdown using siRNA. Compared with the si-NC group, cell migration and invasion was significantly decreased in the si-LAMC2 group. (D, A) WB was used to observe the changes in EMT progression in cal-27 cells following an LAMC2 knockdown using siRNA. Similarly, the EMT process in the si-LAMC2 group significantly differed from that in the si-NC group. (E–G) To observe the changes in cal-27 cell viability and proliferation after an LAMC2 knockdown, we used a clone formation assay and cell proliferation assays. The results were consistent with the above experiments, and cell proliferation and viability were significantly decreased in the si-LAMC2 group. Each experiment was conducted at least three times and at least three sets of data were collected from each experiment for statistical analysis (*p < 0.05; **p < 0.01; ***p < 0.001).

Small interfering RNA (siRNA) was used to knockdown LAMC2 to observe how LAMC2 affects OSCC proliferation, metastasis, and invasion. First, WB was performed to verify the efficiency of LAMC2 downregulation. Three siRNA sequences were used to silence LAMC2. All three sequences successfully silenced the level of LAMC2 protein expression in Cal-27 cells (Fig. 2A). From the three sequences, the sequence (si-LAMC2-1919) with the highest silencing efficiency was selected for subsequent experiments. RT-qPCR and WB were performed to verify the efficiency of LAMC2 downregulation. Fig. 2B showed that the level of gene and protein expression of LAMC2 was successfully knocked down (Fig. 2B). In the wound healing assays and Transwell assay, LAMC2 downregulation inhibited cell metastasis and invasion (Figs. 2C and 2D). Fig. 2E clearly shows that compared with the control group, an important marker of EMT, E-cadherin, was significantly increased. Moreover, Vimentin protein expression, which accelerates the EMT process by changing the cell shape and movement, and Snail, which regulates the EMT process, were significantly decreased [26]. The WB further verified that downregulation of LAMC2 resulted in significant changes to EMT-related proteins and inhibited EMT (Fig. 2E). A clone formation assay and cell proliferation assay verified that the proliferative ability of OSCC was significantly decreased after LAMC2 downregulation (Figs. 2F and 2G). The above experiments demonstrated that the ability of LAMC2 migration, invasion, and proliferation was significantly decreased after silencing in Cal-27 cells. Therefore, LAMC2 plays an active role in OSCC.

### Downregulation of LAMC2 may regulate the PI3K/AKT/mTOR pathway to activate autophagy

**Figure 3:**
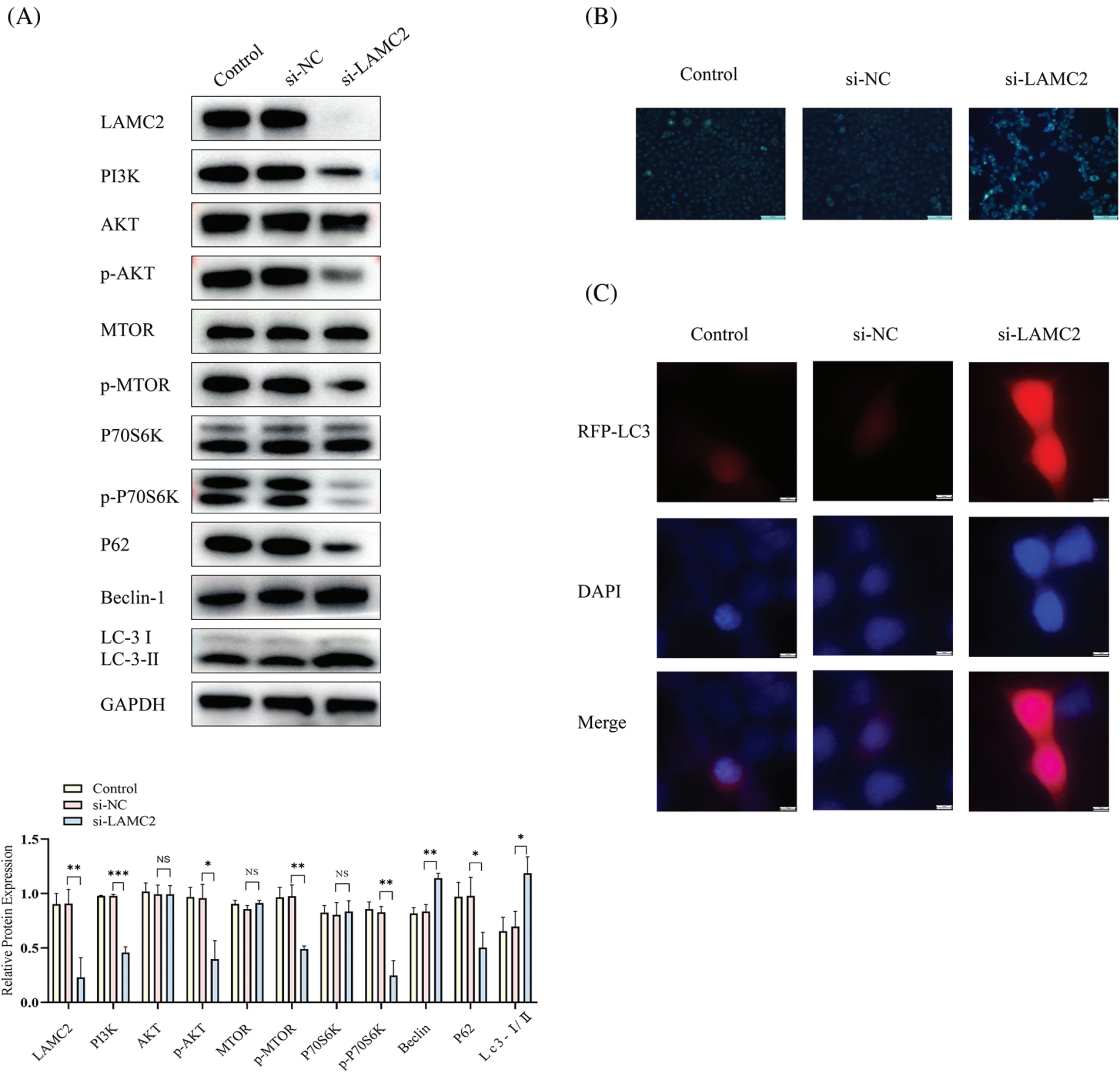
Silencing LAMC2 can activate autophagy through the PI3K/AKT/mTOR pathway. (A) After a knockdown of LAMC2, the changes in the proteins downstream of LAMC2 were displayed by WB. The level of PI3K, AKT, and mTOR protein phosphorylation was significantly decreased, and autophagy-related proteins (LC3, Beclin-1) were activated. B–C show the change in the level of autophagy following LAMC2 downregulation using an MDC staining assay and RFP-LC3. In the si-LAMC2 group, both experiments exhibited increased levels of autophagy. Each experiment was conducted at least three times and at least three sets of data were collected from each experiment for statistical analysis (*p < 0.05; **p < 0.01; ***p < 0.001, NS, not significant).

A WB was used to detect the expression of LAMC2, PI3K, AKT, mTOR, and P70S6K and the degree of phosphorylation of AKT, mTOR, and P70S6K in each group (Fig. 3). The above-mentioned proteins were significantly decreased in the si-LAMC2 group compared to the si-NC group (Fig. 3A). This indicates that LAMC2 regulates downstream signaling through the PI3K/AKT/mTOR pathway. In addition, some changes in autophagy-related proteins were observed when LAMC2 was down-regulated. Fig. 3A shows that in the si-RNA-LAMC2 group, the level of Beclin-1 and LC-3-II was increased, whereas the levels of P62 protein expression were decreased, indicating an active state of autophagy (Fig. 3A) [27,28]. Therefore, we performed a MDC staining assay and observed autophagy flux with RFP-LC3 to further validate our findings. The MDC results showed that the number of autophagosomes increased after the downregulation of LAMC2 (Fig. 3B). Similarly, the RFP-LC3 results displayed an increase in autophagic flux in the si-LAMC2 group compared with that of the si-NC group (Fig. 3C). These results demonstrate that LAMC2 interacts with autophagy via the PI3K/AKT/mTOR signaling pathway.

### Autophagy regulates the biological behavior of OSCC

**Figure 4:**
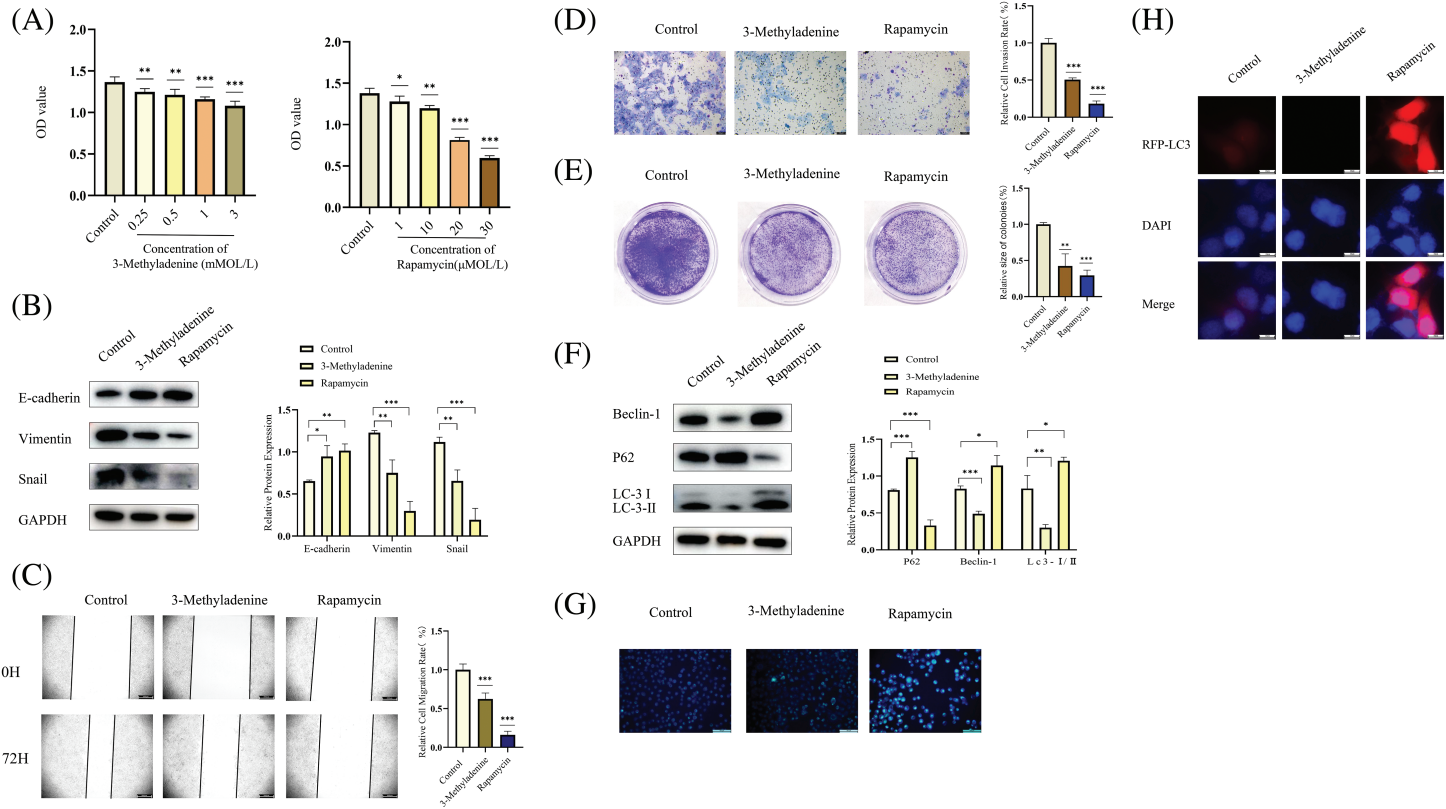
Inhibition or activation of autophagy can inhibit OSCC. (A) A CCK-8 assay was performed to research the effects of different concentrations of 3-Methyladenine and Rapamycin on cell viability. Compared with the control group, different drug concentrations of 3-Methyladenine (0.25, 0.5, 1, 3 mMOL/L) and Rapamycin (1, 10, 20, and 30 μMOL/L) inhibited the viability of Cal-27 cells. (B, F) The changes in autophagy-related proteins and EMT-related proteins were detected by WB after the addition of various drugs. After the addition of 3-Methyladenine (0.5 mMOL/L) and Rapamycin (20 μMOL/L), EMT, and autophagy-related proteins showed corresponding changes. (C–E) The changes in the biological behavior of CAL-27 cells were detected by the Wound healing assays (original magnification: ×50), Transwell invasion (original magnification: ×100), and colony formation assay after the addition of drugs (3-Methyladenine (0.5 mMOL/L) and Rapamycin (20 μMOL/L)). (G–H) show the change in the level of autophagy after the addition of agents (3-Methyladenine (0.5 mMOL/L) and Rapamycin (20 μMOL/L)). The intensity of autophagy increased following the addition of 3-Methyladenine (0.5 mMOL/L). After the addition of Rapamycin (20 μMOL/L), the autophagy intensity was decreased. Each experiment was conducted at least three times and at least three sets of data were collected from each experiment for statistical analysis (*p < 0.05; **p < 0.01; ***p < 0.001).

We used 3-Methyladenine and Rapamycin to investigate the effect of autophagy on the cellular proliferation, metastasis, and invasion of the OSCC tumor. As an autophagy inhibitor, 3-Methyladenine can effectively inhibit autophagy [29]. Rapamycin is also commonly used as an activator of autophagy [30]. First, we used a CCK-8 assay to research the effects of different concentrations of 3-Methyladenine and Rapamycin on cell viability. Interestingly, we found that both 3-Methyladenine (0.25, 0.5, 1, and 3 mMOL/L) and Rapamycin (1, 10, 20, and 30 μMOL/L) inhibited the cell viability of OSCC (Fig. 4A). Combined with the WB experiment, we selected an appropriate concentration for 3-Methyladenine (0.5 mMOL/L) and Rapamycin (20 μMOL/L), respectively, for the following experiment (Fig. 4B). Next, we found that both autophagy activation and inhibition could block the metastasis, invasion and proliferation of OSCC (Figs. 4C–4E). As shown in Fig. 4F, the autophagy related proteins, LC3 and Beclin-1, were increased whereas P62 was decreased, which showed that we successfully activated and inhibited autophagy using 3-Methyladenine or Rapamycin, respectively (Fig. 4F). In addition, we performed an MDC staining assay and RFP-LC3 combined with Fig. 4B to further confirm the changes in autophagy following the addition of 3-Methyladenine and Rapamycin (Figs. 4G and 4H). These results suggest that autophagy plays a complex role in OSCC.

### 3-Methyladenine inhibits autophagy activation induced by LAMC2 downregulation

**Figure 5:**
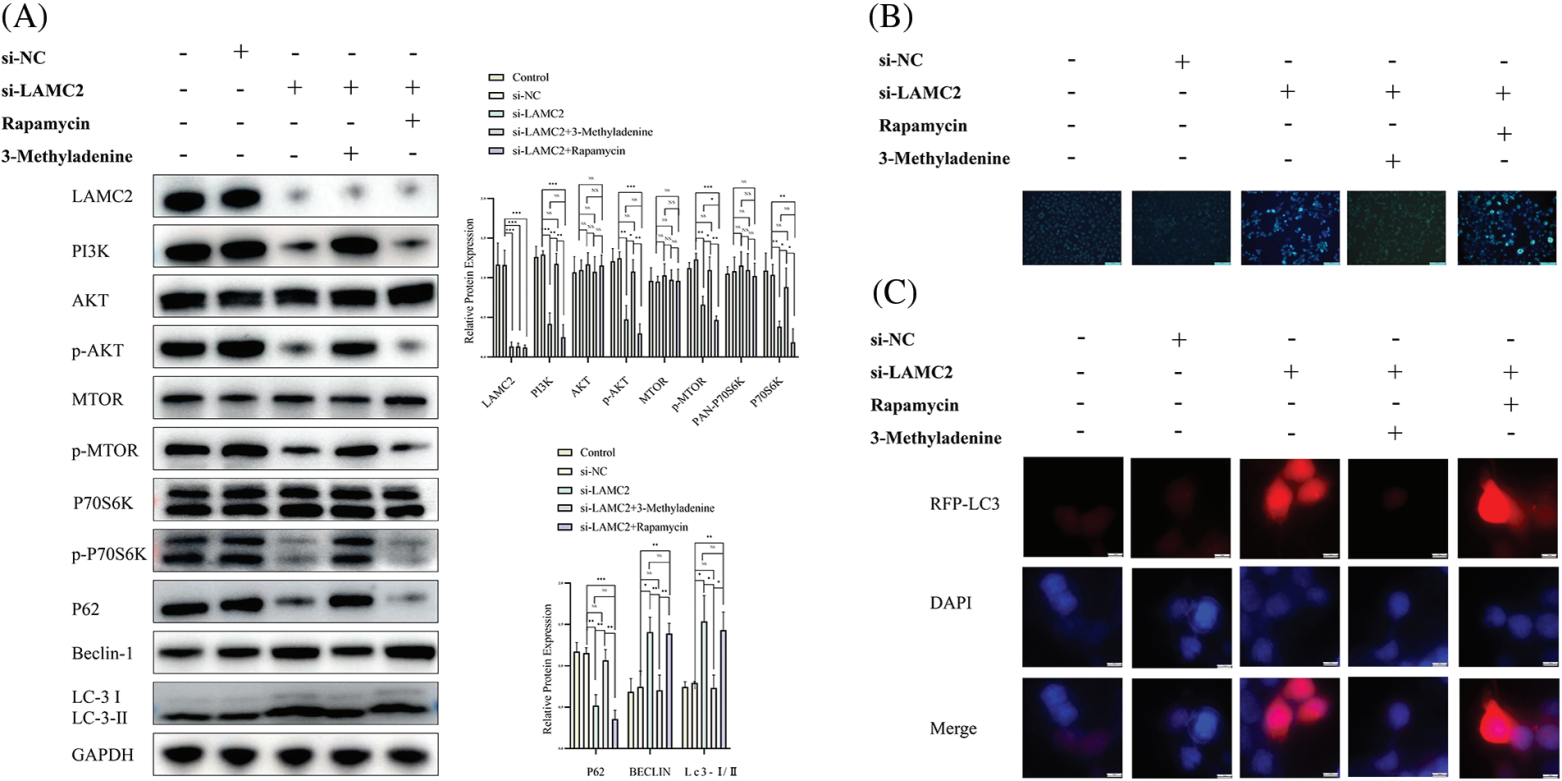
The increased level of autophagy in OSCC induced by LAMC2 silencing was reversed by 3-Methyladenine. (A) After a knockdown of LAMC2 and addition of various drugs (3-Methyladenine (0.5 mMOL/L) and Rapamycin (20 μMOL/L)), the changes in the proteins downstream of LAMC2 were evaluated by WB. The levels of PI3K, AKT, and mTOR protein phosphorylation was significantly decreased in the si-LAMC2 group. Some autophagy-related proteins (LC3, Beclin-1) were activated and other autophagy related proteins (P62) were inhibited. In the si-LAMC2+3MA group, the phosphorylation level of the PI3K, AKT, and mTOR protein signaling pathway and autophagy-related proteins (LC3, Beclin-1, and P62) returned to normal levels. (B, C) show the changes in the level of autophagy following LAMC2 downregulation and the addition of drugs (3-Methyladenine (0.5 mMOL/L) and Rapamycin (20 μMOL/L)) by MDC Staining Assay and RFP-LC3. The results were consistent with the protein results. Each experiment was conducted at least three times and at least three sets of data were collected from each experiment for statistical analysis (*p < 0.05; **p < 0.01; ***p < 0.001, NS, not significant).

To further explore the relationship between LAMC2 and autophagy, we added 3-Methyladenine and Rapamycin based on the downregulation of LAMC2 and its effect on the signaling pathway was observed. In comparison with the si-LAMC2 group, the level of AKT, mTOR, and P70S6K protein of PI3K pathway and phosphorylation in the si-LAMC2 group was decreased by the downregulation of LAMC2 were reversed by 3-Methyladenine in the si-LAMC2+3-Methyladenine group (Fig. 5A). As expected, the above-mentioned proteins decreased by downregulation of LAMC2 were further decreased by Rapamycin in si-LAMC2+ Rapamycin group (Fig. 5A). Moreover, the results showed that neither 3-Methyladenine nor Rapamycin had any effect on LAMC2 at the protein level (Fig. 5A). This is the same result as previously published [31]. We used 3-Methyladenine to reduce the level of increased autophagy induced by the downregulation of LAMC2 back to normal levels to achieve autophagy homeostasis. Combined with the MDC staining assay, the WB analysis, and RFP-LC3, we also found that the activation of autophagy in OSCC induced by the downregulation of LAMC2 in the si-LAMC2 group was also reversed by 3-Methyladenine in the si-LAMC2+3-Methyladenine group (Figs. 5B and 5C). Similarly, we found that the activation of autophagy in OSCC induced by the downregulation of LAMC2 was also further enhanced by Rapamycin in the si-LAMC2 +Rapamycin group (Figs. 5B and 5C). These results also suggest that LAMC2 may interact with autophagy via the PI3K/AKT/mTOR pathway in OSCC.

### Prolferation, metastasis, and invasion decreased by the downregulation of LAMC2 were reversed by 3-Methyladenine

**Figure 6:**
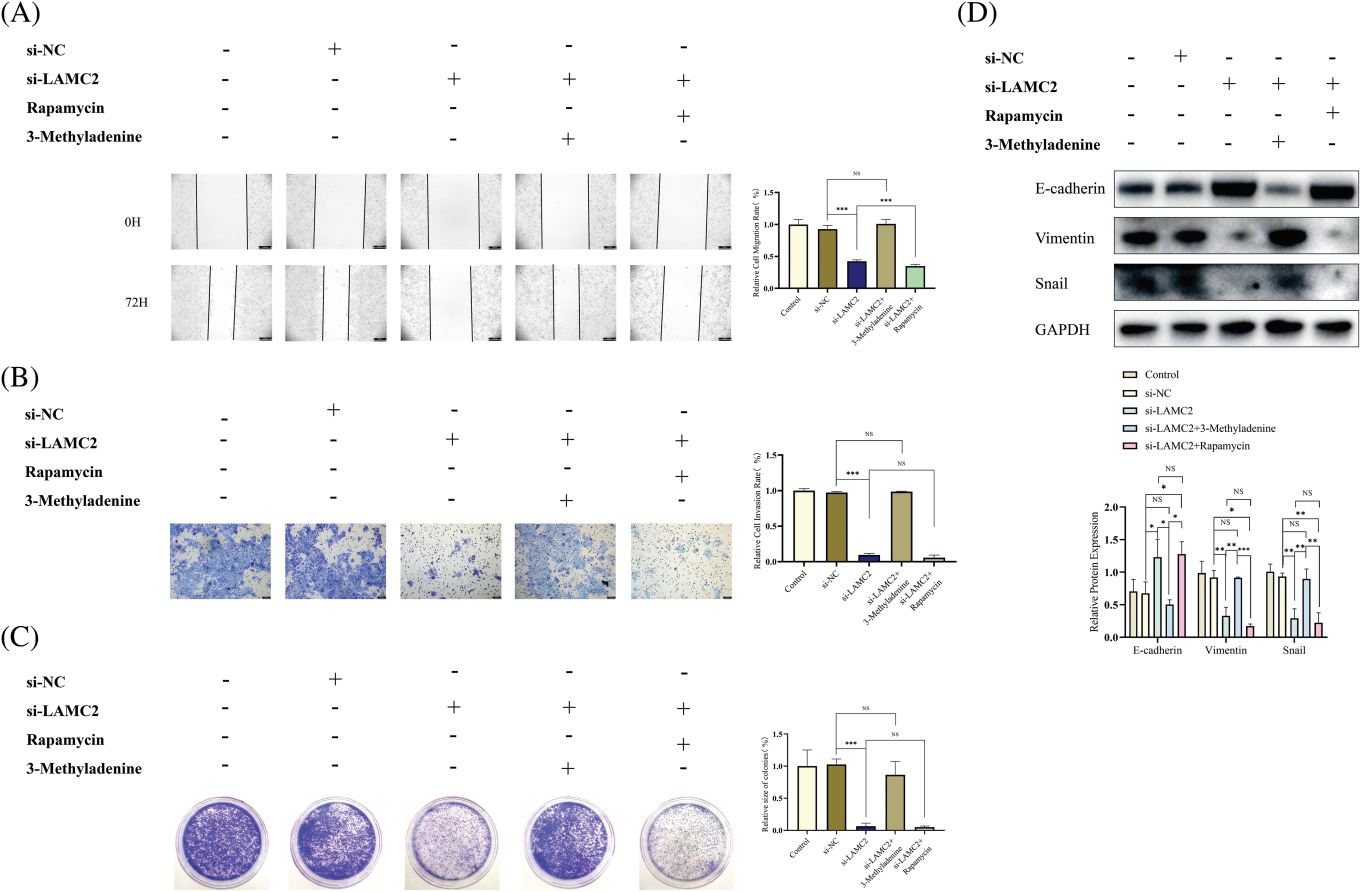
The inhibitory effect of LAMC2 downregulation on OSCC was reversed by 3-Methyladenine. (A–C) Changes in the biological behavior of Cal27 cells were detected by wound healing assays (original magnification: ×50), Transwell invasion (original magnification: ×100) and clone formation assay after the knockdown of LAMC2 and addition of drugs (3-Methyladenine (0.5 mMOL/L) and Rapamycin (20 μMOL/L). (D) WB shows the changes in EMT-related proteins after a knockdown of LAMC2 and addition of drugs (3-Methyladenine (0.5 mMOL/L) and Rapamycin (20 μMOL/L)). Each experiment was conducted at least three times and at least three sets of data were collected in each experiment for statistical analysis (*p < 0.05; **p < 0.01; ***p < 0.001, NS, not significant).

To further explore the relationship between LAMC2, autophagy, and the biological behavior of OSCC, we performed a WB analysis, a clone formation assay, Transwell metastasis assay, and wound-healing assay. As expected, 3-Methyladenine was used to reduce the increased autophagy induced by LAMC2 downregulation to normal levels to achieve autophagy homeostasis. In comparison with the si-LAMC2 group, the ability of proliferation, metastasis, and invasion decreased by LAMC2 downregulation was reversed by 3-Methyladenine in the si-LAMC2+3-Methyladenine group (Figs. 6A–6C). The level of proliferation, metastasis, and invasion in the si -LAMC2+Rapamycin group was further decreased by Rapamycin (Figs. 6A–6C). Moreover, EMT-related proteins also showed the same changes as described above. In the si-LAMC2 group, E-cadherin protein expression was increased and Vimentin and Snail protein expression was decreased. In the si-LAMC2+3-Methyladenine group, the change in protein was reversed by 3-Methyladenine (Fig. 6D). The above experiments further demonstrate that LAMC2 can interact with autophagy to regulate the biological behavior of OSCC.

### LAMC2 plays a positive role in the growth of OSCC in vivo

**Figure 7:**
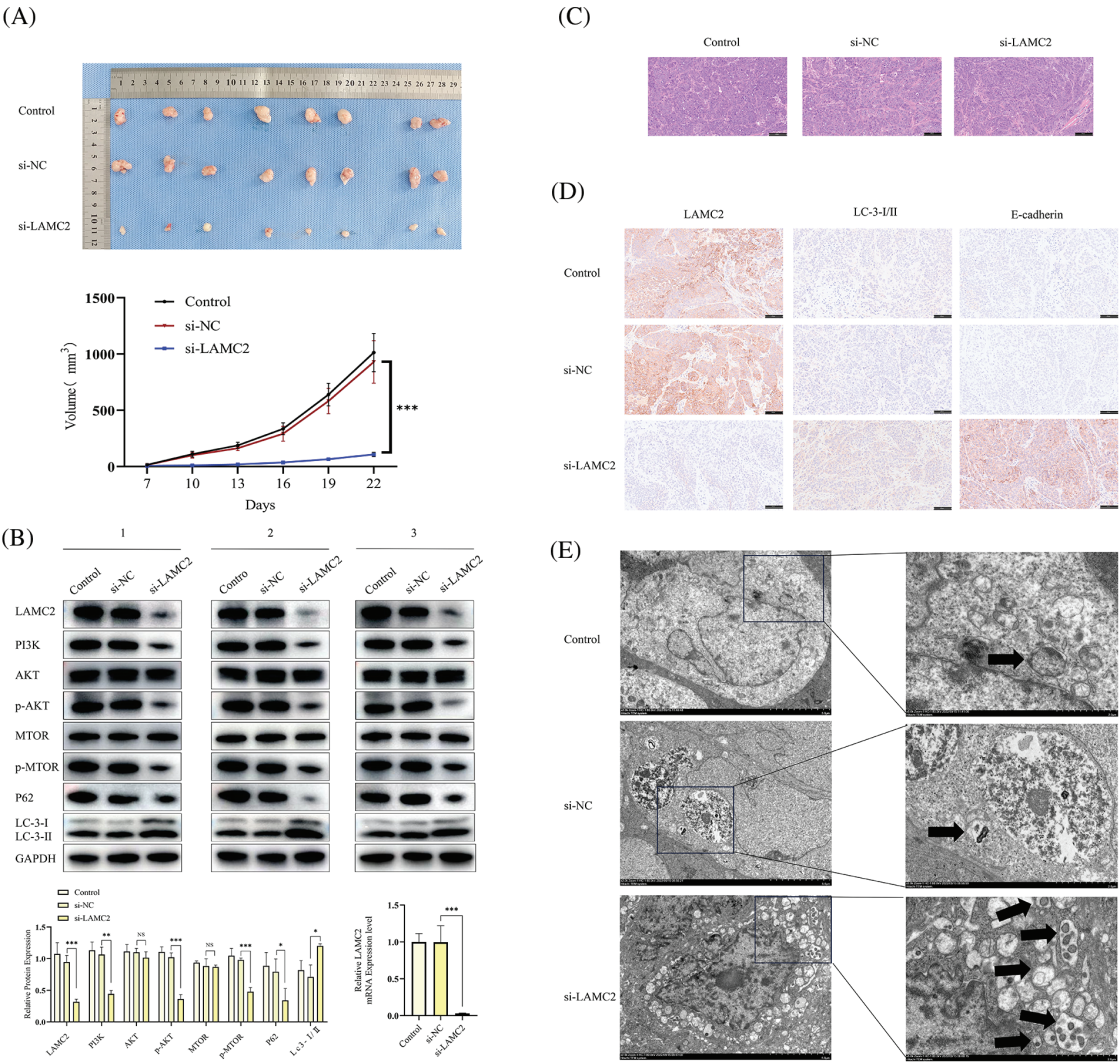
Silencing LAMC2 can increase the level of autophagy in OSCC and inhibit OSCC growth *in vivo*. (A) Tumors were excised from mice and the curve for subcutaneous tumor growth volume was calculated. (B) WB shows the level of different protein expression *in vivo* after a LAMC2 knockdown (three mice were selected from each group). Representative HE staining and IHC results are presented in (C, D). (E) Three sets (Control, si-NC, si-LAMC2) of TEM images (autophagosomes are marked with) (Original magnification: ×2000 and ×5000). Each experiment was conducted at least three times and at least three sets of data collected for statistical analysis for each experiment (*p < 0.05; **p < 0.01; ***p < 0.001, NS, not significant).

To determine the role of LAMC2 in OSCC growth *in vivo*, a cell line-derived xenograft (CDX) model was constructed. Five-week-old Balb/C nude mice were randomly divided into three groups (Control, si-NC, si-LAMC2) with 8 mice per group. Each mouse received 5 × 10^6^ cells/100 μL subcutaneously in the anterior axillary area. The injected cells were stable LAMC2-downregulated expression cells obtained in Section 2.4. When the mice developed visible tumors, the tumor volume was measured every three days (volume = length × width^2^/2). The results showed that the downregulation of LAMC2 inhibited OSCC growth *in vivo* (Fig. 7A). To verify the effect of LAMC2 on PI3K/AKT/mTOR signaling and autophagy-related protein expression *in vivo*, tumor tissues in the animals were detected by WB and RT-qPCR. The *in vivo* PI3K/AKT/mTOR signaling and autophagy-related protein expression was as expected (three mice were selected from each group). In the si-LAMC2 group, the degree of PI3K, AKT, and mTOR protein phosphorylation was significantly inhibited, indicating that the PI3K/AKT/mTOR signaling pathway was inhibited *in vivo* (Fig. 7B). Fig. 7C shows the HE staining of tumor tissue. According to the results of HE experiment, we successfully constructed an OSCC animal CDX model (Fig. 7C). We further verified protein expression in the tissues by IHC. In the si-LAMC2 group, the level of LAMC2 protein expression was significantly decreased, whereas the level of LC3 and E-cadherin protein expression was significantly increased. These results were consistent with WB in animal tumor tissues (Fig. 7D). Moreover, TEM was used to further confirm the number and microstructure of autophagosomes *in vivo*. The results showed an increased number of autophagosomes formed in the si-LAMC2 group (Fig. 7E). This further demonstrated that LAMC2 downregulation activates autophagy through the PI3K/AKT/mTOR signaling pathway.

## Discussion

OSCC is a malignant tumor that threatens human health worldwide. Although there are many sophisticated methods of treating OSCC, the mortality rate remains extremely high. Therefore, there is a requirement for more effective treatments for OSCC. In this study, the downregulation of LAMC2 inhibited the biological behavior of OSCC. This is consistent with the published paper [4]. Moreover, we also found that autophagy could be activated following downregulation of LAMC2 and affected the biological behavior of OSCC. Since the importance of LAMC2 for the treatment of OSCC is obvious, we further explored the mechanism by which LAMC2 regulates the biological behavior of OSCC.

In this study, we regulated autophagy and found that both increased or decreased autophagy had the same effect on OSCC proliferation, metastasis, invasion, and EMT process; however, the extent of the effect differed. Therefore, we sought to determine the effect of autophagy on OSCC.

Autophagy is widely present in cells [32] as it functions to clear intracellular excreta, and autophagy under normal conditions promotes cellular survival (protective autophagy). When cells are stimulated to cause excessive autophagy (cytotoxic/nonprotective autophagy), the cells themselves will cleave the normally functioning organelles and proteins. In contrast, when autophagy is excessively inhibited, there is an accumulation of damaged organelles and waste proteins, both of which break intracellular autophagy homeostasis and have adverse effects on cell viability [22].

Previous studies have shown that autophagy is a highly complex process regarding the regulation of tumors. Autophagy has been suggested to suppress tumors because alleles of the essential autophagy gene, Beclin-1 (ATG6), are lost in some human breast, ovarian, and prostate cancers [33,34]. Another autophagy-related gene, P62, plays a role in cancer. In a mouse model, a P62 deficiency inhibited the development of lung cancer and tumorigenesis triggered by hepatic autophagy deficiency [35–37]. In contrast, compared with normal human tissue cells that survive under normal conditions, the basal level of autophagy in tumor tissue cells that require macronutrient support is abnormally elevated. This indicates that tumor cells activate autophagy to obtain sufficient nutrients [38,39] and autophagy plays a complex role in cancer.

Our findings have shown that either increased or decreased levels of autophagy can inhibit OSCC proliferation, metastasis, and invasion. In untreated tumor cells, autophagy is in a state of homeostasis, and when stimuli is added that exceeds the ability to compensate, disrupting this homeostasis can impact the cellular state and biological behavior. However, an increased level of autophagy caused by LAMC2 downregulation can use drugs to return the level of autophagy to the baseline level in OSCC, reversing the inhibitory effect of breaking autophagy homeostasis induced by tumor biological behavior (Fig. 6).

Unfortunately, considering the complex effects of most autophagy-regulating drugs on the immune system [40,41], *in vivo* experiments based on autophagy regulation were not performed in this study. A more in-depth understanding of the specific role of autophagy in the development of each tumor and the complex biological roles between genes, autophagy and drugs will help develop more effective and less toxic treatments for cancer patients. Due to the complex role of autophagy in tumors, few drugs are available for clinical application. With the development of some novel and high-tech technologies, the combination of clinical and scientific research will definitely provide more effective treatment programs in the future.

**Figure 8:**
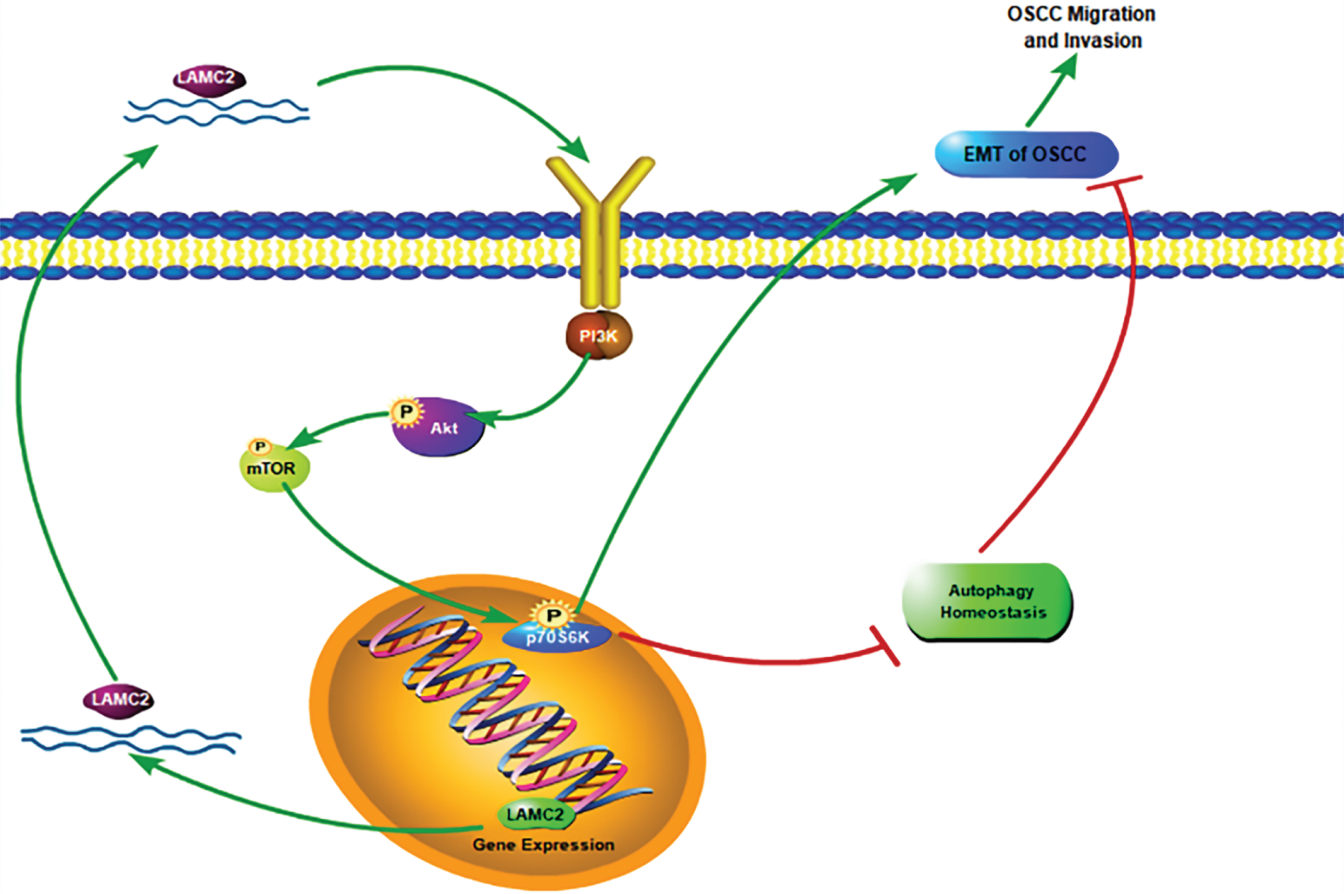
A schematic diagram of LAMC2, autophagy, and the PI3K/AKT/mTOR pathway in OSCC.

In conclusion, our findings demonstrate that LAMC2 regulates autophagy homeostasis and influences themetastasis, invasion, and proliferation of OSCC through the PI3K/AKT/mTOR pathway (Fig. 8). In addition, an interesting phenomenon was observed. The downregulation of LAMC2 activates autophagy and inhibits OSCC proliferation, metastasis, and invasion. As mentioned above, the inhibition and activation of autophagy have the same effect. LAMC2 downregulation can decrease tumor proliferation, metastasis, and invasion, as well as simultaneously inhibit tumor proliferation, metastasis, and invasion by disrupting autophagy homeostasis. Interestingly, when LAMC2 was overexpressed by plasmid transfection on tumor cells with high LAMC2 expression, it had a beneficial effect on the biological behavior of OSCC through the PI3K/AKT/mTOR pathway. However, at the same time, it can also disrupt autophagy homeostasis and adversely affect the biological behavior of tumors. Which of the two plays a more dominant role in this regard is worth exploring (Fig. 8). Therefore, the downregulation of LAMC2 in patients with OSCC combined with autophagy drugs may represent an effective strategy for the clinical treatment of OSCC.

## Data Availability

The datasets used and/or analyzed during the current study are available from the corresponding author on reasonable request.

## Abbreviations

HNSC: Head and neck squamous cell carcinoma
OSCC: Oral squamous cell carcinoma
HNOEC: Human Normal Oral Epithelial Cells
SCC: Squamous cell carcinoma
PI3K: Phosphatidylinositol 3-kinase
p70S6K: Ribosome S6 protein kinase
AKT: Protein kinase B
EMT: Epithelial-mesenchymal transition
IHC: Immunohistochemistry
LC3: Microtubule-associated protein 1 light chain 3
mTOR: Mammalian target of rapamyoin
PCR: Polymerase chain reaction
RT-qPCR: Real time quantitative PCR
siRNA: Small interfering RNA
WB: Western blot
PMSF: Phenylmethanesulfonyl fluoride
PVDF: Polyvinylidene fluoride
CDX: Cell line-derived xenograft
NS: Not significant
